# Saphenous vein graft thrombus findings by scanning electron microscopy in a patient with acute myocardial infarction

**DOI:** 10.1590/S1679-45082013000300024

**Published:** 2013

**Authors:** Marcela Dias Borges, André Haraguti Aguillera, José Joaquim Brilhante, Adriano Caixeta

**Affiliations:** 1 Escola Paulista de Medicina, Universidade Federal de São Paulo, São Paulo, SP, Brazil.; 2 Hospital Israelita Albert Einstein, São Paulo, SP, Brazil.

**Figure 1:**
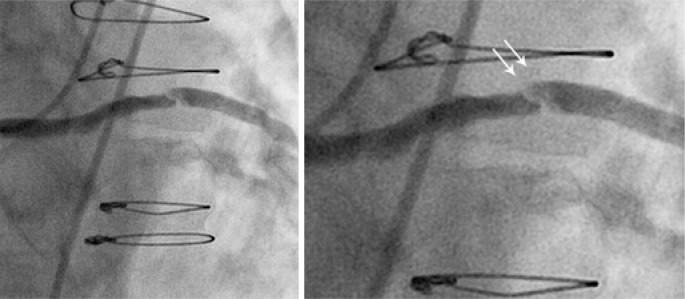
Angiography showing a 90% lesion in saphenous vein graft mid portion

**Figure 2:**
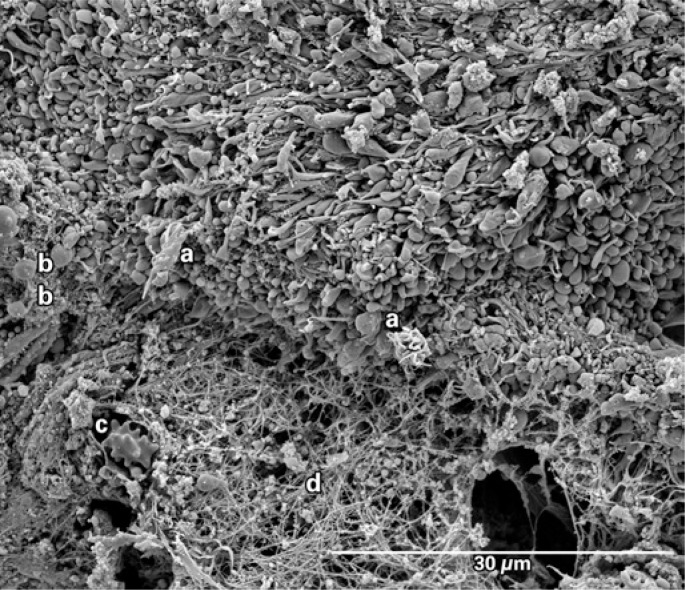
Scanning electron microscopy showing the complex cellular elements of the aspirated material. Notice a predominance of activated platelet aggregates (a) and, on the upper part of the picture, a few platelets partially activated presenting as small oval disks with a few projections or pseudopods (b). An isolated granulocyte (c) and a white fibrin network occupy the lower part of the picture (d)

**Figure 3:**
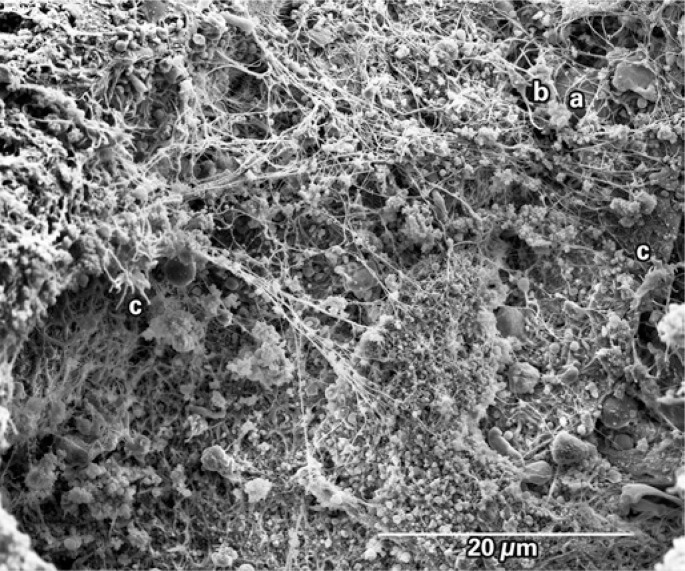
Scanning electron microscopy showing a white fibrin network occupying all the studied area. Note an erythrocyte entrapped under a fibrin network (a), activated platelets near this erythrocyte (b), as well as other activated platelets that are more distant (c)

**Figure 4:**
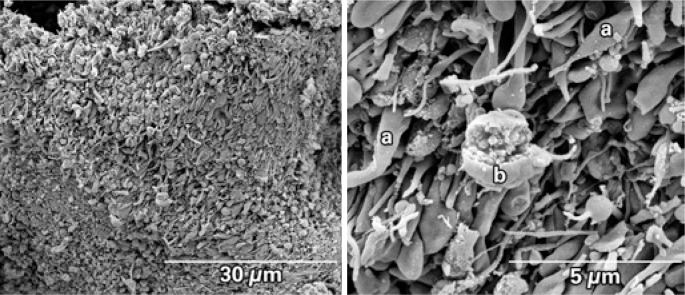
Scanning electron microscopy showing an area with a predominance of platelet aggregates (left). The picture on the right side shows a central area with magnification of 27,221x. A predominance of activated platelets with extending projections or pseudopods (a) is observed. In the central part one leukocyte can be seen (b)

**Figure 5:**
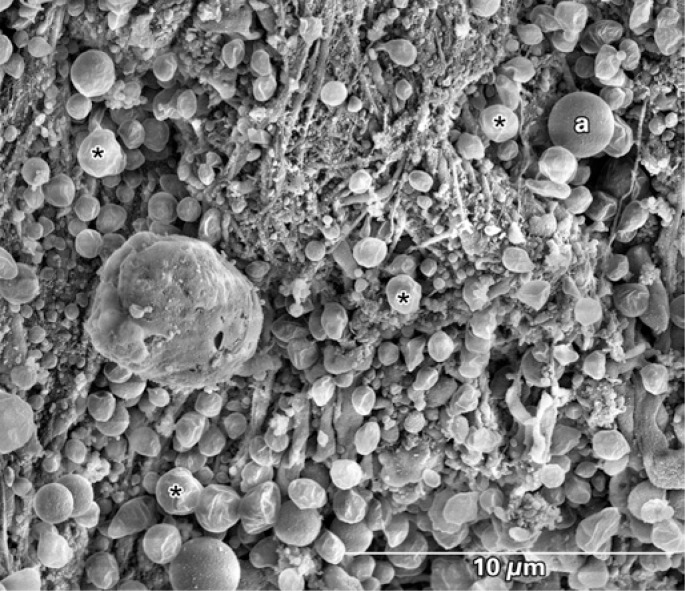
Scanning electron microscopy of the aspirated material. A few fibrin filaments are observed. On the figure's upper part a few erythrocytes are seen (a) and a large quantity of round structures similar to microspheres characteristic of cholesterol crystals (asterisks) can be seen

An eighty-year-old male patient with a history of prior (19 years) coronary artery bypass graft surgery was admitted to the hospital with non ST-segment elevation myocardial infarction (NSTEMI). During the hospital stay he was taking acetylsalicylic acid 100mg per day, a loading dose of 600mg clopidogrel, and low molecular weight heparin 1mg/kg twice a day. Twenty-four hours later the patient underwent coronary angiography, which showed a 90% obstruction in the mid portion of the saphenous vein graft to obtuse marginal with signs of degeneration and local thrombus (Figure 1). Thrombus aspiration was performed with a 6-Fr Export^™^ catheter (Medtronic, Santa Rosa, CA, USA), which removed small reddish colored fragments. They were fixed in 2,5% glutaraldehyde in a 0.1M sodium cacodilate buffer. The material was processed following the GOTO protocol in which the fragments were washed with osmium tetroxide and titanic acid, after which they were dried in a critical-point device and a golden bath. Scanning electron microscopy and high definition photos (3,000 to 27,221x magnification) were obtained by the FEI Quanta^™^ FEG SEM device (FEI Company, Hillsboro, OR, USA). The images showed that the thrombus was rich in activated platelets, with few erythrocytes or inflammatory cells. Many cholesterol crystals were observed (Figures 2 to 5). The fibrin networks were sparse and thin, which is compatible with a short ischemic time and recent thrombus formation.

The recent use of catheter aspiration in STEMI patients allows *in vivo* study of thrombus composition as well as its architectural and dynamic formation. Acute coronary thrombosis is caused by a rupture or erosion of an atherosclerotic plaque with subsequent aggregation of unstable platelets, fibrin networks with entrapped erythrocytes and inflammatory cells. The dynamic process of thrombus formation seems to be related to ischemic time, to the plaque's anatomical underlying substrate and to clinical variables^(1)^. By scanning electron microscopy, Silvain et al. reported the composition of intracoronary thrombi showing that ischemic time in patients with STEMI had a positive correlation with fibrin content and a negative correlation with platelet content^(2)^. In the present case, the first thus far to describe the composition of the thrombus aspirated from a degenerated saphenous vein graft by scanning electron microscopy, the findings of great amounts of smalldimension cholesterol crystals (<10μm) is remarkable, substantially differing from the thrombus composition in native coronary arteries^(2,3)^ – in which the finding of cholesterol crystals is rare. This finding may explain, at least in part, the higher prevalence and greater severity of distal embolization phenomena found in percutaneous intervention in saphenous vein grafts.
